# Plant Glycobiology—a diverse world of lectins, glycoproteins, glycolipids and glycans

**DOI:** 10.3389/fpls.2014.00604

**Published:** 2014-11-04

**Authors:** Nausicaä Lannoo, Els J. M. Van Damme, Cécile Albenne, Elisabeth Jamet

**Affiliations:** ^1^Department of Molecular Biotechnology, Ghent UniversityGhent, Belgium; ^2^Laboratoire de Recherche en Sciences Végétales, UMR 5546, Université Paul Sabatier-Toulouse 3/CNRSCastanet-Tolosan, France

**Keywords:** arabinogalactan proteins, cell wall, hydroxyproline-rich glycoproteins, glycans and glycoconjugates, glycoengineering, lectins, protein-carbohydrate interactions, sugar signaling

Glycosylation is essential for the growth, development or survival of every organism (Varki and Lowe, 2009). Defects in glycan signaling often lead to abnormal development and severe diseases. Glycosylation is ubiquitous and the tremendous structural complexity of glycans makes it quite impossible to predict the biological importance of individual structures. Nowadays, glycans are no longer regarded solely as an energy reservoir, but are associated with storage and transfer of biological information as part of a highly complicated multidimensional coding system (Rüdiger and Gabius, 2009; Gabius et al., 2011; Solís et al., 2014). Plants synthesize a wide variety of unique glycan structures and glycan-binding proteins which play pivotal roles during their life cycle. The increasing number of excellent publications, both in primary and applied plant glycobiology research, demonstrates the great promise and importance of this area for current and future plant science. With 13 original contributions, this Research Topic is a nice compilation of Mini Reviews and Reviews, an Original research paper, and an Opinion Article, highlighting important aspects of plant glycobiology.

In plant glycobiology, *N*-glycans constitute core structures which are grafted on polypeptide backbones. Complex *N*-glycans are ubiquitously present in plants (Wilson et al., 2001), yet their biological function is virtually unknown. Nguema-Ona et al. (2014) provide an overview of the biosynthesis of *N*-glycans. Maeda and Kimura nicely review the group of free *N*-glycans that are released from misfolded proteins or originate from fully processed and secreted proteins by the action of the *N*-glycan releasing enzymes ENGase and PNGase. They discuss the impact of these plant complex *N*-glycans in terms of plant development and fruit ripening (Maeda and Kimura, 2014). The paper from Strasser continues this discussion and focuses on recent developments with respect to *N*-glycan signaling in transgenic *A. thaliana* and rice plants with disabled *N*-glycan processing, which ultimately could lead to the development of some new glyco-engineering tools (Strasser, 2014). Next to *N*-glycans, photosynthesis-derived small sugars such as sucrose, fructose, glucose, trehalose, and derived oligosaccharides, which are generally accepted to be involved in plant energy metabolism and plant growth, have very recently been suggested to act as signal molecules in important plant developmental programs (Ruan, 2014; Smeekens and Hellmann, 2014). In his Opinion Article, Van den Ende (2014) focuses on this intimate communication between plant hormones and small sugars, better-known as the sugar sensing mechanism, and the putative role of small sugars in apical dominance.

Plant cell walls are formed of complex interlaced networks of polysaccharides (cellulose, hemicelluose and pectins) and hydroxyproline-rich *O*-glycoproteins (HRGPs) which are considered as structural proteins (Carpita and Gibeaut, 1993). However, the way these macromolecules are arranged in supramolecular scaffolds is still poorly understood. Knoch et al. (2014) focus on the recent discoveries of carbohydrate active enzymes (CAZy) (Lombard et al., 2014) that are involved in the synthesis as well as in the degradation of arabinogalactan proteins (AGPs), i.e., a highly diverse class of cell surface HRGPs found in most plant species. They discuss the role of these enzymes in plant development. Nguema-Ona et al. (2014) and Hijazi et al. (2014) broaden this discussion and present an overview of the enzymes not only involved in the synthesis of AGPs, but also of extensins, another type of HRGPs, and discuss the importance of both AGPs and extensins for proper cell wall development and morphology as well as their role in biotic stress responses. Hijazi et al. (2014) propose a new model to explain how all types of HRGPs could contribute to a continuous glyco-network with their respective partners including polysaccharides to form a complex architecture in plant cell walls. In the case of secondary cell walls, lignin, and different types of hemicelluloses are found. Hao et al. (2014) present an Original Research paper in which they identified a galacturonosyltransferase (GAUT12) from *A. thaliana* as a new glycosyltransferase possibly contributing to the synthesis of a polysaccharidic structure including pectins allowing the deposition of xylan and lignin.

Plant cell walls not only have a structural function, but also play a critical role in the perception of invading pathogens and the activation of specific plant defense responses, as discussed by Lannoo and Van Damme (2014). This review elaborates how plants can recognize plant pathogens or predators upon perception of characteristic epitopes or damage-associated patterns, using protein-protein interactions as well as protein-glycan interactions mediated by lectins. In addition, they highlight that protein-glycan interactions mediated by different types of nucleocytoplasmic lectins are part of signaling pathways implicated in plant defense responses. Plant lectins not only attracted a lot of attention due to their phytoprotective properties, they are also of interest for medical applications and use in biomedical diagnosis. They can be purified from natural resources, but with the increasing demand for biopharmaceuticals, different expression platforms are being exploited for their recombinant production. Oliveira et al. (2014) describe how they can produce recombinant frutalin, a lectin from *Artocarpus incisa* (breadfruit) which possesses immuno-modulatory, anti-tumor, and tumor biomarker properties, in distinct microbial systems. Since the presence and quality of glycosylation plays a crucial role for the pharmacological properties of the therapeutic protein, also plants have received growing attention for molecular farming. In this Research Topic, several papers review the humanization of the plant glycosylation pathway allowing the production of human proteins with optimized glycosylation profiles in eukaryotic microalgae (Mathieu-Rivet et al., 2014), lower plants (mosses) (Decker et al., 2014) and in higher plants (De Meyer and Depicker, 2014; Loos and Steinkellner, 2014).

The major aim of this Research Topic was to provide the reader an overview of the latest progress in plant glycobiology research. All contributions demonstrate recent and exciting breakthroughs and present the intrinsic capacity of this particular scientific research area for further improvement of plant biotechnology. We hope that this e-book can provide useful information to readers and stimulate future research in the dynamic plant glycobiology community.

## Conflict of interest statement

The authors declare that the research was conducted in the absence of any commercial or financial relationships that could be construed as a potential conflict of interest.
